# Fast Neuronal Calcium Signals in Brain Slices Loaded With Fluo‐4 AM Ester

**DOI:** 10.1111/ejn.16657

**Published:** 2025-01-13

**Authors:** Ömer Yusuf İpek, Fatima Abbas, Hajar Sajidy, Marco Canepari

**Affiliations:** ^1^ Université Grenoble Alpes, CNRS, LIPhy Grenoble France; ^2^ Institute of Health Sciences/Physiology Erciyes University Kayseri Turkey; ^3^ Department of Physiology, Faculty of Medicine Kirşehir Ahi Evran University Kirşehir Turkey; ^4^ Laboratories of Excellence Ion Channel Science and Therapeutics Valbonne France; ^5^ Institut National de la Santé et Recherche Médicale Paris France

**Keywords:** brain slices, calcium imaging, experimental epilepsy research, hippocampal slices

## Abstract

Staining brain slices with acetoxymethyl ester (AM) Ca^2+^ dyes is a straightforward procedure to load multiple cells, and Fluo‐4 is a commonly used high‐affinity indicator due to its very large dynamic range. It has been shown that this dye preferentially stains glial cells, providing slow and large Ca^2+^ transients, but it is questionable whether and at which temporal resolution it can also report Ca^2+^ transients from neuronal cells. Here, by electrically stimulating mouse hippocampal slices, we resolved fast neuronal signals corresponding to 1%–3% maximal fluorescence changes. Specifically, by recording Ca^2+^ fluorescence at 2000 frames/s from multiple sites both in the CA3 and in the CA1 regions, we observed that the signal measured near the stimulating electrode, positioned on the mossy fibre pathway, was not blocked by perfusion with 10 μM NBQX and 50 μM AP5, preventing excitatory synaptic transmission. In contrast, this signal was fully blocked by additional perfusion with 1 μM tetrodotoxin, inhibiting voltage‐gated Na^+^ channels and neuronal action potentials. We also present recordings obtained in the presence of 10 μM of the GABA_A_ receptor antagonist bicuculline, or of 50 μM of the voltage‐gated K^+^ channel inhibitor 4‐aminopyridine, exhibiting a wide propagation of the signal from CA3 to CA1 regions under conditions that mimic epileptic seizures. Altogether, while Fluo‐4 AM remains a preferable indicator for reporting Ca^2+^ signals from astrocytes at slow temporal resolution, we demonstrated that it can be also utilised for analysing fast neuronal network activity elicited by electrical stimulation in brain slices.

## Graphical abstract

We show that the commonly used high‐affinity calcium indicator Fluo‐4 AM, routinely utilised to record slow calcium signals from glial cells, is also suitable to resolve fast calcium transients from neurons. We demonstrate the feasibility of a new approach, using this dye for calcium imaging at high spatial and temporal resolution, which allows analysing stimulated neuronal network activity elicited in brain slices.

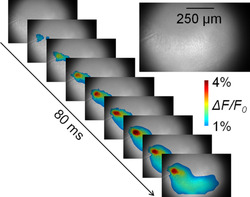

**Abbreviations**
AM: acetoxymethyl
AMPAR: AMPA receptors
AP: action potential
AP‐5: DL‐2‐amino‐5‐phosphonopentanoic acid
KS: Kolmogorov–Smirnov
MF: mossy fibre
NBQX: 2,3‐dioxo‐6‐nitro‐1,2,3,4‐tetrahydrobenzo[*f*]quinoxaline‐7‐sulfonamide
NMDAR: NMDA receptors
SNR: signal‐to‐noise ratio
SO: stratum oriens
SR: stratum radiatum
TTX: tetrodotoxin
VGCC: voltage‐gated Ca^2+^ channel
4‐AP: 4‐aminopyridine (4‐AP)
ΔF/F_0_
: fractional change of fluorescence
ΔF/F_0_^max^
: maximum of ΔF/F_0_

## 1 Introduction

Fluo‐4 is a high affinity Ca^2+^ indicator (Kd~345 nM, Gee et al. 2000) that is widely used to stain a variety of cells and tissues (Paredes et al. 2008), being characterised by large dynamic range (Thomas et al. 2000). When using the cell permeant form where the fluorophore is attached to an acetoxymethyl (AM) group, the molecule can penetrate the plasma membrane and, once inside the cell, the endogenous esterases cleave the AM group releasing the dye that accumulates in the cytosol. With this approach, many cells can be simultaneously stained and, theoretically, all cells can be loaded in the same manner. In practice, the staining of different cell types is not uniform. When Fluo‐4 AM was used to stain thalamic brain slices, a preferential loading for glial cells was observed (Parri, Gould, and Crunelli 2001; Parri and Crunelli 2003). This allowed measuring large and slow Ca^2+^ waves from astrocytes with 0.8–5‐s temporal resolution in the presence of the sodium channel inhibitor tetrodotoxin (TTX) that blocks the onset of neuronal action potentials (APs). Several other studies have confirmed this preferential glial signalling using Fluo‐4 AM both in slices (see, e.g. Dallwig and Deitmer 2002; Zur Nieden and Deitmer 2006) or in vivo (see, e.g. Hirase et al. 2004; Hoogland, Kuhn, and Wang 2011), but also these studies focussed on slow signals on a 1‐s time scale. Yet, whether and at what extent Fluo‐4 AM can be also used to measure neuronal Ca^2+^ transients in brain slices remained an elusive question. An important aspect to consider is that indicators of the Fluo family are dim in the absence of free Ca^2+^ (Oertner et al. 2002). As a consequence, the dynamic range of Fluo‐4 is extremely high (Thomas et al. 2000) and it is expected that cells with higher resting Ca^2+^ would be brighter. Notably, it has been shown that resting Ca^2+^ in principal hippocampal neurons is significantly lower than that in the surrounding astroglia (Zheng et al. 2015). This evidence suggests that the observed higher fluorescence in glial cells can be also partially attributed to higher levels of free Ca^2+^. Given that glial cells dominate the background fluorescence in brain slices, a consequence is that fractional changes in fluorescence (ΔF/F_0_) associated with neuronal Ca^2+^ transients are likely to be much smaller compared to those from glial cells. Thus, from a methodological perspective, it is important to test whether fast neuronal Ca^2+^ signals, arising from Ca^2+^ influx through voltage‐gated Ca^2+^ channels (VGCCs) during neuronal firing or through Ca^2+^‐permeable ionotropic synaptic receptors during synaptic transmission, can be effectively measured in brain slices.

In this technical note, we provide evidence of Ca^2+^ fluorescence transients originating from neurons in mouse hippocampal slices stained with Fluo‐4 AM esters. To achieve this result, we recorded fluorescence at high spatial resolution (> 0.5 MP) with 0.5 ms frame interval from large hippocampal areas, including the CA3 and the CA1 regions, during and after stimulation of the mossy fibre (MF) pathway. In all slices tested, a train of six stimuli at 100 Hz elicited a ΔF/F_0_ signal of 1%–3% on the MF pathway and in adjacent regions. This signal was reduced, but not fully blocked, by addition of glutamate receptor antagonists while the residual signal was fully blocked by TTX. The signal was enhanced by bicuculline or 4‐aminopyridine (4‐AP) (Ives and Jefferys 1990). Thus, we illustrate how this Ca^2+^ indicator can be used to explore neuronal network signals in brain slices.

## 2 Material and Methods

### 2.1 Ethics Statement

Experiments in brain slices were performed at the Laboratory of Interdisciplinary Physics in Grenoble in accordance with European Directives 2010/63/UE on the care, welfare and treatment of animals. Procedures were reviewed by the ethics committee affiliated to the animal facility of the university (E38421100001).

### 2.2 Slice Preparation, Solutions, Fluo‐4 Staining and Pharmacology

Transversal (horizontal) hippocampal slices were prepared from 30‐ to 40‐postnatal‐day‐old C57Bl6 mice (of both genders) using a Leica VT1200 (Leica, Wetzlar, Germany) as previously described (Jaafari, De Waard, and Canepari 2014; Jaafari, Marret, and Canepari 2015; Jaafari and Canepari 2016; Filipis et al. 2018). In this study, the thickness of the slice was set to 350 μm to maximise the preservation of connectivity while maintaining the slices in good health. The extracellular solution contained (in millimolar) 125 NaCl, 26 NaHCO_3_, 20 glucose, 3 KCl, 1 NaH_2_PO_4_ and 2 CaCl_2_ bubbled with 95% O_2_ and 5% CO_2_. After slicing, all slices were preincubated for 45 min at 37°C and then maintained at room temperature. The procedure of staining with Fluo‐4 was similar to that used in the past for Fluo‐3 AM esters (Mammano et al. 2000; Canepari et al. 2000). Specifically, individual 50‐μg vials of Fluo‐4 AM ester (purchased either from Thermo Fisher Scientific or from Biotium, Fremont, California) were predissolved at 5 mM in DMSO. Each slice was incubated at room temperature in a solution containing 2–3 μM of the dye for 30–45 min. To avoid potentially toxic effects, we did not use Pluronic F‐127 that is commonly utilised to improve dye loading in this type of staining procedures (Hamad, Krause, and Wahle 2015). The following pharmacological agents (purchased from HelloBio, Dunshaughlin, Republic of Ireland) were used. To antagonise glutamate AMPA receptors (AMPAR), we used 10 μM 2,3‐dioxo‐6‐nitro‐1,2,3,4‐tetrahydrobenzo[*f*]quinoxaline‐7‐sulfonamide (NBQX) disodium salt dissolved in H_2_O. To antagonise glutamate NMDA receptors (NMDAR), we used 50 μM DL‐2‐amino‐5‐phosphonopentanoic acid (AP5) dissolved in H_2_O. To prevent activation of voltage‐gated Na^+^ channels (VGNCs) mediating neuronal APs, we used 1 μM TTX dissolved in H_2_O. To antagonise GABA_A_ receptors, we used 10 μM bicuculline initially dissolved at 100 mM in DMSO. To inhibit voltage‐gated K^+^ channels, we used 50 μM 4‐AP dissolved in H_2_O.

### 2.3 Ca^2+^ Imaging

**FIGURE 1:**
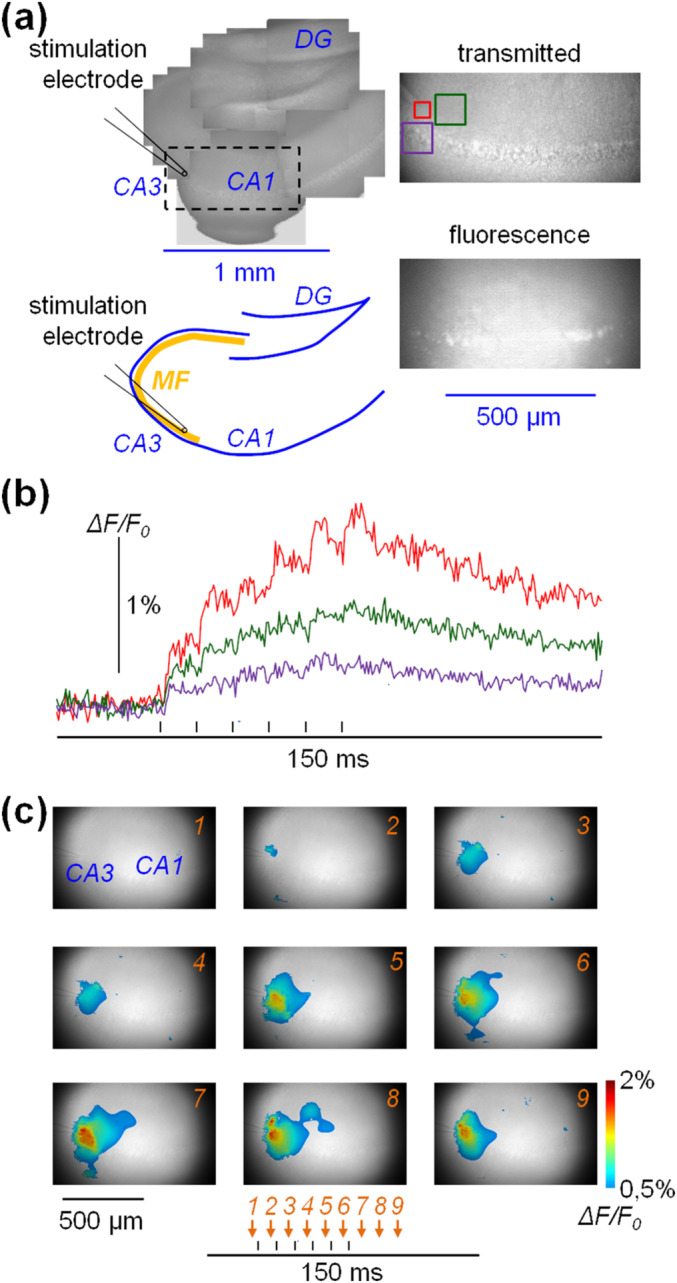
Imaging Fluo‐4 Ca^2+^ transients elicited by MF stimulation. (a) Left, reconstruction of the hippocampus by multiple transmitted‐light images (top) and schematics of the structures (bottom); the positions of the stimulating electrode over the MF pathway and the recording area (dashed rectangle) are indicated. Right, recording area under transmitted light (top) and fluorescence (bottom). Three square sites over the transmitted‐light image are indicated; red: stimulating site; green and purple: 100 μm from the stimulating site in the SR and SO respectively. The timing of the stimulating pulses is indicated below by the vertical lines. (b) ΔF/F_0_ signals produced by six stimulating pulses at 10‐ms interval in the square sites reported in (a). (c) In another slice, the ΔF/F_0_ signal in nine frames of the sequence, with 10‐ms interval between two consecutive frames, filtered as described in the Materials and Methods section, is depicted using a colour scale over the image under transmitted light. Values below 0.5% are not shown. The timing of each frame (1–9) with respect to the stimulating protocol is indicated below by the arrows.

After Fluo‐4 staining, slices were transferred into the recording system and continuously perfused with extracellular solution at 32°C–34°C. The system was based on an Olympus BX51 microscope equipped with a 25×/1.05 NA objective (model XLPLN25XWMP2). Fluorescence was excited at 470 nm with an OptoLED (Cairn Research, Faversham, United Kingdom) and recorded at 530 ± 21 nm using a Kinetix (Teledyne Photometrics, Tucson, Arizona) CMOS camera at 2000 frames/s and 8‐bit digital depth. Before camera acquisition, the image was demagnified by 0.25×. The ability of this imaging system to record from large hippocampal areas is illustrated in Figure 1a. The field of the objective covering an area of ~720 μm and a reconstruction of the hippocampus, obtained by multiple images under transmitted IR light sampled at 16‐bit depth, is shown on the top‐left side. The area underlined by the dashed‐line rectangle corresponds to the position of the recording region shown on the right. Images under transmitted light at 16 bits (top) and under Fluo‐4 fluorescence (bottom) at 8 bits were composed, in this example, of 770 × 353 pixels, with each pixel corresponding to ~1 μm. In this study, fluorescence images of up to 900 × 650 pixels could be acquired at 2000 frames/s at 8‐bit depth. Pipettes of 2–4‐μm diameter filled with extracellular solution, positioned on the end of the stratum lucidum corresponding to the last part of the MF pathway, were used as stimulating electrodes. The stimulation protocol consisted of six current pulses of 10–40‐μA amplitude and 100‐μs duration delivered at 100 Hz during a trial lasting 200 ms.

### 2.4 Data Analysis

Data were analysed using custom‐written code in MATLAB. Frame sequences, either from single trials or from averages of two to four trials (when these trials gave identical responses), were corrected for photobleaching by subtracting the frame sequence from a trial without stimulation. Signals were expressed as ΔF/F_0_ signals. When averaged in defined regions, a Savitzky–Golay smoothing filter (Savitzky and Golay 1964) was applied to the trial without stimulation to minimise the additional noise introduced by bleaching correction. In this analysis, the maximum of ΔF/F_0_ (ΔF/F_0_^max^) was extracted from each experiment under control conditions and/or in the presence of pharmacological agents. When producing the colour‐scaled frames and videos, a mask was applied to remove dark pixels and a 41‐pixel two‐dimensional average filter was applied to each frame before calculating the ΔF/F_0_ signal. These were further filtered three times using an 80‐pixel two‐dimensional Wiener filter, and ΔF/F_0_ frames were illustrated using a colour scale superimposed to an image under IR transmitted light sampled at 16‐bit digital depth. In the three animations online (Movies S1–S3), each frame was the average of four original frames and movies were run at 10 frames/s. The colour scale was not reported in these videos.

### 2.5 Statistical Analysis

This study comprises experiments from *N* = 37 slices. In seven slices, measurements were performed sequentially in control conditions, after perfusion with NBQX and AP5 and after further perfusion with NBQX, AP5 and TTX. In one slice, measurements were performed sequentially in control conditions and after perfusion with bicuculline. In one slice, measurements were performed sequentially in control conditions and after perfusion with 4‐AP. In all other slices, measurements were performed at one pharmacological condition only. Statistical results are reported as mean ± SD. In the paper, *p* values obtained in statistical tests were referred as significant when *p* < 0.01, but the exact values of the tests, as well as the results from individual slices, are reported in the “Metafile” available online in the public repository Zenodo (https://zenodo.org/records/13365448). The effects of NBQX and AP5, or the effect of TTX in the presence of NBQX and AP5, were assessed using single trials, and we performed recordings within 10 min after bath perfusion with pharmacological agents to prevent signal rundown. In these experiments, the effect of pharmacological agents was tested by performing a paired *t*‐test under control conditions and after perfusion with NBQX and AP5, or in the presence of NBQX and AP5 and after further addition of TTX. To test differences between experiments performed in control solution or in the presence of bicuculline or of 4‐AP, a Kolmogorov–Smirnov (KS) test was performed. Since these experiments were only exploratory, *p* values should be interpreted accordingly.

## 3 Results

### 3.1 Spatial and Temporal Profile of  Fluo‐4 Ca^2+^ Transient Elicited by MF Stimulation

The initial challenge in this technical study was to quantify ΔF/F_0_ signals with adequate signal‐to‐noise ratio (SNR). This was consistently achieved by averaging fluorescence over regions of 50 × 50 pixels or 100 × 100 pixels, obtaining the summation of enough photons necessary to measure ΔF/F_0_ transients > 0.2%. Figure 1b shows a representative example, from *N* = 17 slices under control conditions, of ΔF/F_0_ signals from the three colour‐outlined squares illustrated in Figure 1a. In the red 50 × 50 pixel rectangle, centred over the tip of the stimulating electrode and corresponding to the last part of the MF pathway, the contributions to the ΔF/F_0_ signal from each of the six stimulating pulses were clearly distinguishable. In that region, the ΔF/F_0_ signal reached its maximum of ~1.5% a few frames after the last stimulating pulse. In the 100 × 100 pixel green square, positioned at the beginning of the stratum radiatum (SR) and centred at a distance of approximately 100 μm from the tip of the stimulating electrode, a smaller ΔF/F_0_ signal was observed. At this site, the contributions of individual stimulating pulses were not visually distinguishable. A smaller ΔF/F_0_ signal with similar shape was observed in the 100 × 100 pixel purple square, which spanned the stratum pyramidale and the stratum oriens (SO) also at a distance of ~100 μm from the stimulating electrode tip. A straightforward way to illustrate the spatial and temporal distribution of the ΔF/F_0_ signal across the slice is to depict the signal using a colour scale over a transmitted‐light image of the slice, as shown in the example of Figure 1c. In this example, ΔF/F_0_ values above 2% are depicted with the same dark red tone whereas ΔF/F_0_ values below 0.5% (close to the noise level) are not shown. The signal started after the first stimulating pulse (Frame 2) at a site adjacent to the electrode and spread through surrounding sites in the direction of the CA1 region during the pulse train (Frames 3–7) and beyond (Frames 8–9). Notably, the signal expanded up to ~250 μm from the stimulating electrode without reaching more distal parts of the CA1 region. The evolution of the ΔF/F_0_ signal at reduced time scale before, during and after the pulse train over an interval of 150 ms can be appreciated in Movie S1. The quantitative results from the representative example analysed in this section serve as premise for the detailed characterisation of the signals provided in the next section.

### 3.2 Fluo‐4 Ca^2+^ Transients Elicited by MF Stimulation Are Diminished by Glutamate Receptor Antagonists NBQX and AP5 and Fully Blocked by TTX

**FIGURE 2:**
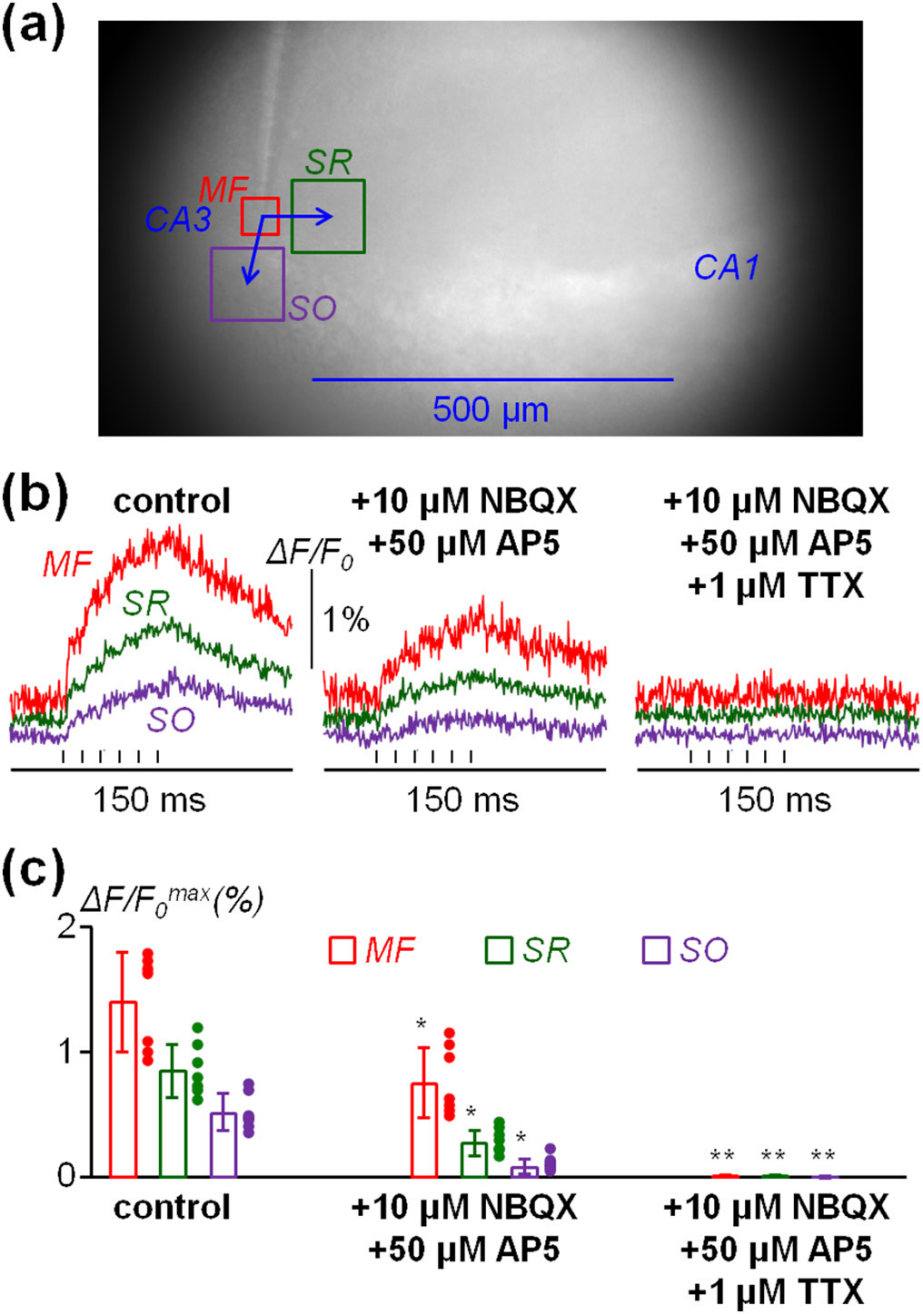
Fluo‐4 Ca^2+^ transients elicited by MF stimulation are reduced by NBQX and AP5 and fully blocked by TTX. (a) Transmitted‐light image of a slice with three square sites indicated the following: the red square (50 × 50 pixels) is centred over the stimulating electrode positioned on the MF pathway; the green square (100 × 100 pixels) is in the SR at 100‐μm distance from the stimulating electrode; the purple square (100 × 100 pixels) is in the SO at 100‐μm distance from the stimulating electrode. (b) ΔF/F_0_ signals produced by six stimulating pulses at 10‐ms interval in the square sites reported in (a) under control conditions (left); after perfusion with 10 μM NBQX and 50 μM AP5 (centre); and after further perfusion with 10 μM NBQX, 50 μM AP5 and 1 μM TTX (right). The timing of the stimulating pulses is indicated below by the vertical lines. (c) Scatter plot of single‐slice values and bars reporting mean ± SD of the Δ*F*/*F*_0_ maximum (Δ*F*/*F*_0_^max^) in MF, SR and SO sites (see (a)) in *N* = 7 slices under control conditions, after perfusion with 10 μM NBQX and 50 μM AP5 and after further perfusion with 10 μM NBQX, 50 μM AP5 and 1 μM TTX. *ΔF/F_0_^max^ in the presence of NBQX and AP5 was significantly smaller than that in control conditions (*p* < 0.01, paired *t*‐test). **ΔF/F_0_^max^ in the presence of NBQX, AP5 and TTX was significantly smaller than that in the presence of NBQX and AP5 only (*p* < 0.01, paired *t*‐test).

**FIGURE 3:**
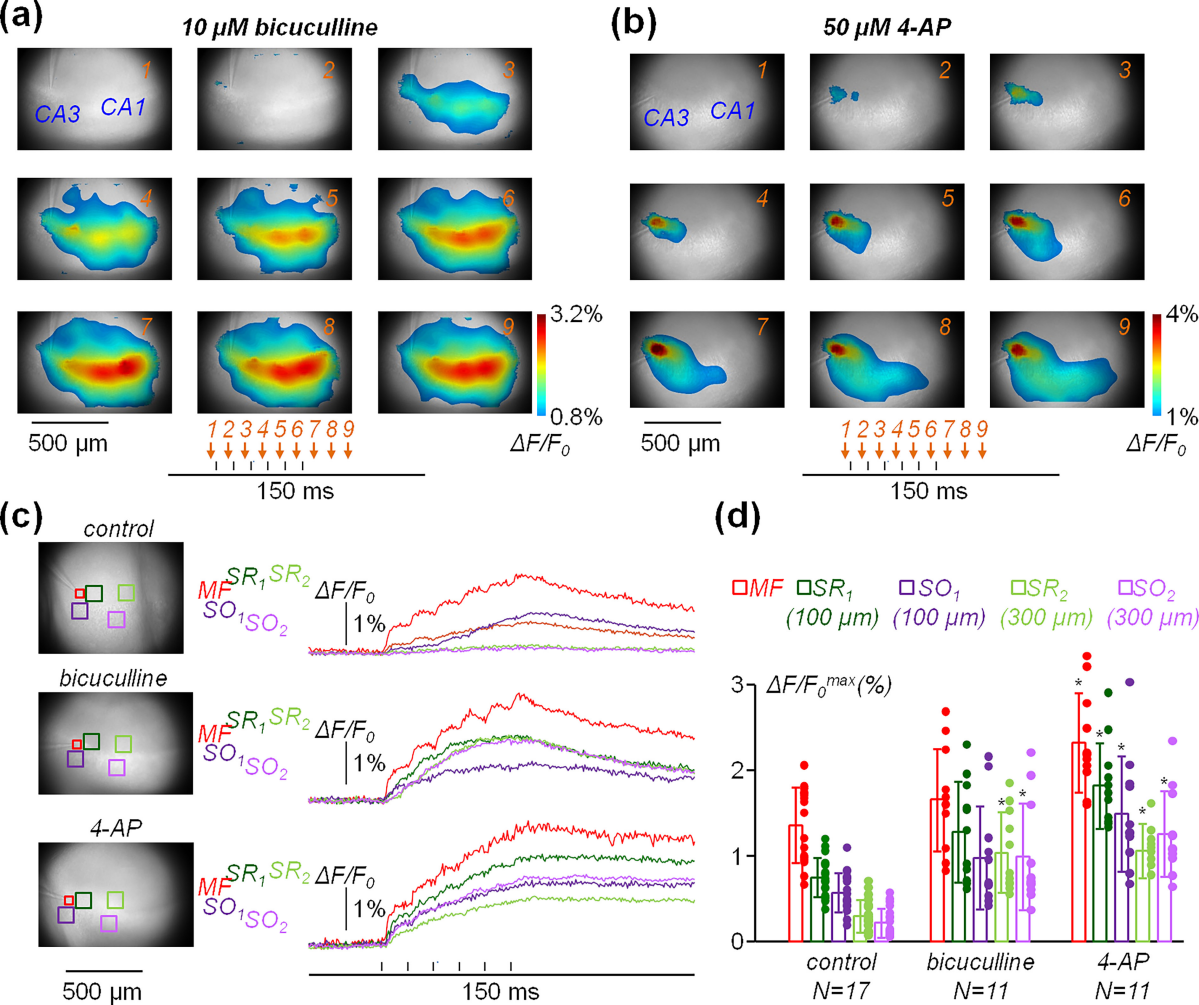
Fluo‐4 Ca^2+^ transients in the presence of bicuculline or of 4‐AP. (a) ΔF/F_0_ signal in nine frames of the sequence, in the presence of 10 μM bicuculline, depicted using a colour scale over the image under transmitted light. The timing of each frame (1–9) with respect to the stimulating protocol is indicated below by the arrows. In this case, values below 0.8% are not shown. (b) In another slice, same as (a), but in the presence of 50 μM 4‐AP. In this case, values below 1% are not shown. (c) Left, transmitted‐light image of three slices with five square sites indicated; the red square (50 × 50 pixels) is centred over the stimulating electrode positioned on the MF pathway (MF); the dark green square (100 × 100 pixels) is in the SR at 100‐μm distance from the stimulating electrode (*SR*_*1*_); the dark purple square (100 × 100 pixels) is in the SO at 100‐μm distance from the stimulating electrode (*SO*_*1*_); the light green square (100 × 100 pixels) is in the SR at 300‐μm distance from the stimulating electrode (*SR*_*2*_); the light purple square (100 × 100 pixels) is in the SO at 300‐μm distance from the stimulating electrode (*SO*_*2*_). Right, ΔF/F_0_ signals produced by six stimulating pulses at 10‐ms interval in the square sites reported on the left. The experiment on the top was performed under control conditions; the experiment in the middle was performed in the presence of 10 μM bicuculline; the experiment on the bottom was performed in the presence of 50 μM 4‐AP. (d) Scatter plot of single‐slice values and bars reporting mean ± SD of ΔF/F_0_^max^ in MF, SR_1_, SO_1_, SR_2_ and SO_2_ sites (see (c)) in *N* = 17 slices under control conditions, in *N* = 11 slices in the presence of 10 μM bicuculline and in *N* = 11 slices in the presence of 50 μM 4‐AP. *ΔF/F_0_^max^ either in the presence of bicuculline or of 4‐AP was significantly larger than that in control conditions (*p* < 0.01, KS test).

The fast signals shown in Figure 1 can originate from different sources, in particular from Ca^2+^ influx via receptors activated by synaptic transmission or via VGCCs activated by neuronal firing. To distinguish between the two sources, we first analysed the effect of antagonising glutamate receptors and then the effect of blocking neuronal APs on the residual Ca^2+^ transient. In the slice illustrated in Figure 2a, we analysed the ΔF/F_0_ signal in the 50 × 50 pixel square centred on the tip of the stimulating electrode and in two 100 × 100 squares at 100 μm from the tip of the stimulating electrode, within the SR and in the SO. As shown in Figure 2b, at the stimulated MF site, the ΔF/F_0_ signal was nearly half of its initial amplitude after perfusion with a solution containing 10 μM of the AMPAR antagonist NBQX and 50 μM of the NMDAR antagonist AP5. The reduction of the ΔF/F_0_ signals in the SR and SO sites was even greater, indicating that in these two areas, the signals under control conditions were primarily driven by synaptic transmission. Further perfusion with a solution also containing 1 μM of the VGNC inhibitor TTX fully blocked ΔF/F_0_ signals in all squares. Altogether, we performed this experiment in *N* = 7 slices where ΔF/F_0_^max^, in MF, SR and SO sites respectively, was 1.40 ± 0.40%, 0.85 ± 0.21% and 0.51 ± 0.15% under control conditions; 0.75 ± 0.28%, 0.27 ± 0.10% and 0.08 ± 0.06% after NBQX and AP5 bath perfusion; and 0.01 ± 0.01%, 0.01 ± 0.01% and 0.00 ± 0.01% after NBQX, AP5 and TTX bath perfusion (Figure 3c). The ratio of ΔF/F_0_^max^ after and before NBQX and AP5 bath perfusion was 0.54 ± 0.11 in the MF region, 0.33 ± 0.13 in the SR region and 0.17 ± 0.10 in the SO region. In all regions, the reduction was significant (*p* < 0.01, paired *t*‐test), and further perfusion with TTX fully blocked the residual signal in all slices tested. These two results demonstrate that a large portion of the Fluo‐4 transient elicited by MF stimulation originates from cells not directly stimulated but targeted by excitatory synaptic transmission. Nevertheless, the substantial contribution that was resistant to the block of synaptic transmission and could be attributed to direct stimulation was fully inhibited by TTX and therefore, it originated exclusively from multiple compartments of neuronal cells that are capable of firing APs.

### 3.3 Fluo‐4 Ca^2+^ Transients Elicited by MF Stimulation Are Enhanced by Bicuculline or 4‐AP Application

To assess how Fluo‐4 can be used to investigate neuronal network activity in hippocampal slices, we pharmacologically induced epileptic conditions by incubating the slice either with 10 μM bicuculline (see, e.g. Ault and Wang 1986; Salgado and Alkadhi 1995; Borck and Jefferys 1999) or with 50 μM 4‐AP (see, e.g. Rutecki, Lebeda, and Johnston 1987; Avoli 1990; Wahab et al. 2010). In the example of Figure 3a, a spatial and temporal distribution of the ΔF/F_0_ signal in the presence of bicuculline is illustrated. In contrast to the example in control conditions reported in Figure 1c, the signal starting at a site adjacent to the stimulating electrode (Frame 2) rapidly spread over the whole observable CA1 region (Frames 3–9). The full evolution of the ΔF/F_0_ signal in this example can be appreciated at reduced time scale in Movie S2. The striking difference in the evoked activity between experiments in control conditions and in the presence of bicuculline can be attributed to the crucial role of feedforward and feedback inhibition in preventing hyperexcitable states (Sloviter 1991). This phenomenon can be particularly observed when slices are cut in horizontal sections (Velazquez and Carlen 1999). In the example of Figure 3b, a spatial and temporal distribution of the ΔF/F_0_ signal in the presence of 4‐AP is illustrated. In this case, the size of the ΔF/F_0_ signal was substantially larger with ΔF/F_0_^max^ > 3% in a relatively large area adjacent to the stimulating electrode. In addition, the spread of the signal was slower and more persistent (see also the full evolution of the ΔF/F_0_ signal reported in Movie S3). The spatial and temporal profile of ΔF/F_0_ signals in the presence of can be attributed to a combination of several effects. As reported in a milestone study (Perreault and Avoli 1992), 4‐AP acts on the firing of GABAergic cells enhancing K^+^ output and therefore promoting network instability. In parallel, 4‐AP acts on dendritic K^+^ channels in pyramidal neurons enhancing cell firing and Ca^2+^ influx via VGCCs (Magee and Carruth 1999). In the three examples of Figure 3c, reporting results from different slices, ΔF/F_0_ signals from five sites are shown. Specifically, in addition to the signal at the stimulating site (*MF*) and at the two sites of the SR and SO at 100 μm from the soma (*SR*_*1*_ and *SO*_*1*_), we analysed two more distal sites of the SR and SO at 300 μm from the soma (*SR*_*2*_ and *SO*_*2*_), in order to quantify the spread of the signal. Under control conditions (example on the top), ΔF/F_0_ transients at *SR*_*2*_ and *SO*_*2*_ were within the noise level, whereas in the examples with bicuculline or with 4‐AP, the signal at these sites was ~1%. Furthermore, while ΔF/F_0_ signals began decaying a few frames after the end of the stimulation in the examples under control condition or with bicuculline, this decay did not occur in the example with 4‐AP. Experiments were performed in *N* = 11 slices in the presence of 10 μM bicuculline and in *N* = 11 slices in the presence of 50 μM 4‐AP, and the results were compared to those in *N* = 17 slices under control conditions. As shown in Figure 3d, ΔF/F_0_ signals above noise level were consistently observed at *SR*_*2*_ and *SO*_*2*_ sites both with bicuculline and with 4‐AP, unlike in experiments under control conditions. In addition, ΔF/F_0_^max^ was significantly larger with 4‐AP, with respect to control conditions, at all sites tested (*p* < 0.01, KS test). Finally, when estimating the decay of the signal at the stimulating site by linearly fitting the signal from its peak to the end of the recording, the slope was −7.7 ± 2.4%/s in control slices (*N* = 17) and −8.5 ± 4.0%/s in the presence of bicuculline (*N* = 11). Both slopes were significantly faster (*p* < 0.01, KS test) with respect to the slope calculated in slices in the presence of 4‐AP (−2.8 ± 2.3%/s, *N* = 11). Although only exploratory, these experiments show the ability of the measurements to discriminate neuronal network behaviours under different conditions.

## 4 Discussion

In this technical note, we report the possibility of recording neuronal Ca^2+^ transients in brain slices stained with the indicator Fluo‐4 AM. When a brain slice is incubated with this molecules for > 30 min and then washed with artificial cerebrospinal fluid, a background fluorescence from the surface, mostly originating in glial cells (Parri, Gould, and Crunelli 2001; Parri and Crunelli 2003), is observed. Since Ca^2+^ transients are measured as fluorescence changes from background fluorescence, large ΔF/F_0_ transients from astrocytes can be measured (Parri and Crunelli 2003). Two important further features of specific glial Ca^2+^ waves, that can also appear spontaneously, make them easily observable: they are slow (in the range of hundreds of milliseconds or seconds), and they propagate through surrounding astrocytes (Charles 1998). Consequently, these glial Ca^2+^ waves are dominant when fluorescence is recorded by slowly scanning the slice. But what happens if fluorescence is recorded at high temporal resolution (in the kilohertz range) following extracellular electrical stimulation? Although the stimulating electrode excites several adjacent neurons that simultaneously elevate their intracellular Ca^2+^, the dominant glial background fluorescence strongly limits the size of the associated neuronal ΔF/F_0_ transient. In addition, if directly excited, neurons release glutamate and Ca^2+^ elevations via NMDAR (and AMPAR) in synaptically activated neurons, and possibly in glial cells expressing the same glutamate receptors, can contribute to the ΔF/F_0_ transient. The targeted neurons may also fire APs spreading the signal beyond the site of the stimulating electrode. The signals outside the stimulated site, however, are sparser and less synchronous, and therefore, ΔF/F_0_ transients are smaller than those near the stimulating electrode. In the present experiments, ΔF/F_0_^max^ was typically 1%–3% at the stimulated MF site, but consistently smaller at ~100 μm from the tip of the stimulating electrode. Finally, unlike slow astrocyte Ca^2+^ waves, which are dominated by contributions from store release (Charles 1998), neuronal Ca^2+^ transients are much faster requiring fluorescence recording at higher temporal resolution. Thus, the possibility of measuring evoked neuronal Ca^2+^ transients ultimately relies on the ability of the imaging system to resolve ΔF/F_0_ signals below 1% at high acquisition rates. In the past, Ca^2+^ transients from hippocampal slices stained with AM indicators could be measured at low spatial resolution (10 × 10 pixels) at 400 frames/s using a photodiode array (Albowitz, König, and Kuhnt 1997) or at higher spatial resolution, but at the reduced rate of 50–100 frames/s using a cooled CCD camera (Murayama et al. 2005). However, CMOS technology offers today superior performance to simultaneously achieve high spatial and temporal resolution (Beier and Ibey 2014). In this study, by reducing the sampling digital depth to 8 bits, we achieved recordings at 2000 frames/s with over 0.5M pixel resolution. Then, by averaging fluorescence from ≥ 2.5K pixels (from areas ≥ 2.5 mm^2^), we could eventually dispose of enough photons to resolve ΔF/F_0_ signals < 0.2% with adequate SNR. This enhanced imaging capability enabled us to conduct an exploratory characterisation of hippocampal network activity under various pharmacological conditions. In conclusion, we show that brain slices stained with Fluo‐4 AM can be in the future exploited to quantitatively study neuronal network dynamics and to investigate the effects of pharmacological agents.

## Author Contributions

**Ömer Yusuf İpek:** data curation, formal analysis, investigation, software. **Fatima Abbas:** investigation, methodology, resources. **Hajar Sajidy:** investigation. **Marco Canepari:** conceptualization, data curation, formal analysis, funding acquisition, investigation, methodology, project administration, resources, software, supervision, validation, visualization, writing – original draft.

## Conflicts of Interest

The authors declare no conflicts of interest.

## Supporting information


**Movie S1** Supporting information.

**Movie S2** Supporting information.

**Movie S3** Supporting information.

**Data S1** Supporting information.

## Data Availability

The dataset comprising frame sequences from *N* = 37 slices, used in this study, is available in the public repository Zenodo (https://zenodo.org/records/13365448).
