# Supplementary material for: Fast Neuronal Calcium Signals in Brain Slices Loaded With Fluo‐4 AM Ester
Source: Eur J Neurosci. 2025 Jan 13;61(1):e16657. doi: 10.1111/ejn.16657 (PMC11727817; doi:10.1111/ejn.16657)
Supplement: Supplementary file 4 — Data S1 Supporting information. [file EJN-61-0-s004.pdf]

Fast neuronal calcium signals in brain slices loaded with Fluo-4 AM ester

Ömer Yusuf Ipek, **Fatima Abbas**, **Hajar Sajidy**, **Thibault Fresneau**, **Marco Canepari**

Supplementary Movies Legend

Colour scale movies report the Ca^{2+} fractional change of fluorescence ($\Delta F/F_0$) associated with MF electrical stimulation in hippocampal slices. The colours scale span: from 0.5% to 2% is Supplementary Movie 1; from 0.8% to 3.2% is Supplementary Movie 2; from 4% to 1% is Supplementary Movie 3.
